# Duty of Notification and Aviation Safety—A Study of Fatal Aviation Accidents in the United States in 2015

**DOI:** 10.3390/ijerph15061258

**Published:** 2018-06-13

**Authors:** Alpo Vuorio, Bruce Budowle, Antti Sajantila, Tanja Laukkala, Ilkka Junttila, Stein E. Kravik, Robin Griffiths

**Affiliations:** 1 Mehiläinen Airport Health Centre, 01530 Vantaa, Finland; 2 Department of Forensic Medicine, University of Helsinki, 00014 Helsinki, Finland; antti.sajantila@helsinki.fi; 3 Center for Human Identification, University of North Texas Health Science Center, 3500 Camp Bowie Blvd., Fort Worth, TX 76107, USA; bruce.budowle@unthsc.edu; 4 Mehiläinen Kielotie Health Centre, 01730 Vantaa, Finland; tanja.laukkala@kela.fi; 5 Faculty of Medicine and Life Sciences, University of Tampere, 33100 Tampere, Finland; ilkka.junttila@uta.fi; 6 Fimlab Laboratories, 33100 Tampere, Finland; 7 NASA’s Ames Research Center, Mountain View, CA 94035, USA; sekravik@gmail.com; 8 Occupational and Aviation Medicine, University of Otago, 6242 Wellington, New Zealand; rob.griffiths@otago.ac.nz

**Keywords:** accountability, duty of notification, aeromedical practitioners, fatal accident, aviation, safety

## Abstract

After the Germanwings accident, the French Safety Investigation Authority (BEA) recommended that the World Health Organization (WHO) and European Community (EC) develop clear rules for the duty of notification process. Aeromedical practitioners (AMEs) face a dilemma when considering the duty of notification and conflicts between pilot privacy and public and third-party safety. When balancing accountability, knowledge of the duty of notification process, legislation and the clarification of a doctor’s own set of values should be assessed a priori. Relatively little is known of the magnitude of this problem in aviation safety. To address this, the National Transportation Safety Board (NTSB) database was searched to identify fatal accidents during 2015 in the United States in which a deceased pilot used a prescribed medication or had a disease that potentially reduced pilot performance and was not reported to the AME. Altogether, 202 finalized accident reports with toxicology were available from (the year) 2015. In 5% (10/202) of these reports, the pilot had either a medication or a disease not reported to an AME which according to the accident investigation was causal to the fatal accident. In addition, the various approaches to duty of notification in aviation in New Zealand, Finland and Norway are discussed. The process of notification of authorities without a pilot’s express permission needs to be carried out by using a guidance protocol that works within legislation and professional responsibilities to address the pilot and the public, as well as the healthcare provider. Professional guidance defining this duty of notification is urgently needed.

## 1. Background

Aeromedical practitioners (AMEs), or medical practitioners, may face a dilemma when considering the duty of notification and possible conflicts between pilot privacy and public safety. Part of the dilemma is created by the differences in a patient’s and/or doctor’s personal values and the profession’s core values, which are conduct defined by legislation. In principle, a doctor should treat patients with mutual understanding, but the patient can decline treatment and therapeutic or diagnostic procedure(s). In all cases, a doctor shall address the situation professionally and accordingly weigh the circumstances. The World Medical Association (WMA) was founded in 1947 [1], and its central objective is to promote the highest possible standards of ethical conduct and care by physicians. In the latest version of the WMA Declaration of Geneva, which was published on 19th March 2018, an addition was accepted that “doctors should respect the autonomy and dignity of their patients”. An individual AME’s dilemma is then to balance accountability in aviation medicine. On the other hand, an AME needs to respect a pilot’s privileges while bearing in mind a duty of notification if the pilot is for some reason not willing to co-operate (e.g., declines suggested tests). Therefore, knowledge of the process, legislation and the clarification of a doctor’s own set of values should be assessed a priori. Moreover, the doctor should appreciate possible fears a patient may have and try to create an environment in which a patient feels protected [2]. While a pilot’s trust is an essential key element, the AME also has wider responsibilities based on medical ethics and legislation to protect and promote both the health of the pilot and public safety.

### 1.1. Special Features from New Zealand, Norway, Finland and United States

New Zealand represents a country with detailed guidance material for duty of notification. Finland is an example in which legislation for duty of notification in aviation currently does not concern all doctors equally, but emphasizes the role of an AME. Norway is an example of a country that has duty of notification in aviation concerning all doctors, opticians and psychologists. In the United States, duty of notification responsibilities are unclear [3]. Reporting responsibilities vary State by State. According to an FAA report [3] “the perceptions of adverse legal consequences of reporting appear to be greater than not reporting”. AMEs have the duty to report issues potentially affecting public safety. Regarding doctors in general, there are concerns violating patient privacy.

While in Europe, or, for that matter, globally, a consensus on the details of an AME’s duty of notification is being discussed, the New Zealand Civil Aviation Authority has implemented legislation regarding any doctor’s duty of notification [4] (Table 1). New Zealand recognized that the requirement of a doctor’s duty of notification should be actively promoted and education for doctors advocated. This view requires attention because the decision by a doctor to notify, which is part of their duty, can be at odds with maintaining the privacy of the pilot. If pilots expect that their privacy will be violated, they may not to seek necessary care.

In New Zealand, a doctor’s duties and rights are defined legislatively. The duties and rights are as follows: “a medical practitioner (1) has reasonable grounds to believe that a person is a license holder and (2) is aware, or has reasonable grounds to suspect, that the license holder has a medical condition that may interfere with the safe exercise of the privileges to which the license holder’s medical certificate relates, the medical practitioner must, as soon as practicable, (1) inform the license holder that the authority will be advised of the condition, and (2) advise the authority of the condition” [4]. An aviation examiner or medical practitioner is not subject to any civil or criminal liability (indemnified) if he/she is carrying out an act by advising, expressing or stating authority. The liabilities are not so clear for a lesser pilot license such as recreational pilot licenses.

In Norway, doctors, psychologists and opticians have had a duty of notification since 1982. On average, the local CAA reports about five notifications annually [5]. In Finland, current duty of notification in transport (excluding AMEs) only covers ground transportation (driver’s licenses). Changes have been suggested to this legislation to cover all doctors and psychologists. The circumstances of recent airplane accidents in Finland have led to these recommendations [5,6]. In 2014, the Finnish Safety Investigation Authority recommended that additional training, especially of specialists, should include the option of consulting the pilot’s AME or the Transport Safety Agency [6]. Last year, the Finnish Safety Agency recommended standardizing duty of notification between aviation and road transport [5].

### 1.2. Germanwings Accident and Safety Recommendations

After the Germanwings accident, the French Safety Investigation Authority Bureau d’Enquêtes et d’Analyses (BEA) recommended that both the World Health Organization (WHO) and European Community (EC) develop clear rules for the duty of notification [7]. These rules should require health care providers to inform the appropriate authorities when a specific patient’s health is likely to impact public safety. The BEA recognized that both the doctor and the patient should be protected in the notification process to increase overall safety. It is expected that this process will be complicated in the EC, because medical confidentiality is not purely a medical matter, but also an important societal concern [8]. In 2016, the European Aviation Safety Authority (EASA) submitted a Working Paper to the European Commission on the issue of balancing patient confidentiality and public safety [9]. While individual health examinations will never be able to prevent all serious aircraft accidents due to health issues, they can likely help reduce such events within the context of pilot privacy and public and pilot safety [10]. The Federal Aviation Authority (FAA) report was based on a study of 6677 pilots fatally injured in accidents in U.S. between 1990 and 2012 [11]. It was noted that there is a need to improve communication between doctors and pilots about the risk that prescribed drugs may cause to aviation performance. The objective of the present study is to identify those fatal accidents in which the drug or disease was a contributory factor of the accident according to National Transport Safety Board (NTSB), and identify those cases in which doctors should have potentially used duty of notification. This information will give an idea of the extent of this problem.

## 2. Methods

The National Transportation Safety Board (NTSB) database was searched [12] to identify all fatal aviation accidents in 2015 in the United States. The search date was 12th February in 2018. 236 accidents were initially identified, and each accident report was analyzed manually (A.V.). Fatal accidents without final report (accident investigation underway) or without post-mortem toxicology, or the pilot being without medical certificate and thus not participating in obligatory heath examinations, were excluded. After these exclusions, 202 accident investigations remained. Fatal aviation accidents, in which a deceased pilot used prescribed medication or had a disease or disorder that was causal or contributory for the fatal accident according to the NTSB and was not known by an aeromedical examiner (AME), were chosen for in-depth analysis. This in-depth analysis was done independently by two authors (A.V., T.L.), and disagreements were resolved by discussion based on re-evaluation of the finalized accident report, and medical, toxicological and autopsy data were analyzed.

## 3. Results

Altogether, 202 finalized accident reports with detailed information on performed autopsies including reported toxicology findings were identified from the year 2015 in U.S. In 5% (0.05) (10/202) of these reports the pilot had not reported medication or a disease which was concluded to have been causal or contributory to the fatal aviation accident according to the NTSB accident investigation (Table 2).

Eight of the accidents were related to general aviation, one was a taxi-flight and one was an agricultural flight. In four out of these ten fatal accidents, amphetamine or methamphetamine had been used by the pilot. In two cases, it remained unclear whether the stimulant was for medical or recreational use. Stimulant use was in each of these cases considered as a contributory factor to the fatal accident by NTSB.

There were two cases of benzodiazepine use. Both pilots used prescription medication alprazolam. Alprazolam use was judged to worsen performance among other flying incompatible medications and was determined to be a contributory cause in the fatal accident of these two pilots.

There was one fatal accident case in which selective serotonin re-uptake inhibitor (SSRI) antidepressant sertraline was present in post-mortem analysis. However, the FAA protocol for its accepted use was not followed [13].

In one fatal accident case, the pilot used quetiapine and hydrocodone. Quetiapine is an antipsychotic prescription medication and is not compatible with flying. Hydrocodone is an opioid prescription painkiller affecting the central nervous system and is not compatible with flying. In this fatal accident, medication was a contributing cause for the accident by impairing performance, according to NTSB.

A recent heart attack was found in one case. The accident was caused by incapacitation due to complications of a recent acute myocardial infarction. Acute myocardial infarction is a major change in pilot’s health, and if pilots themselves do not inform the proper authority, there is a need for a doctor to inform the authority.

Altogether, eight or nine of these ten accidents were related to the pilot’s psychiatric or substance abuse problems and/or related treatments or drug abuse, and one or two out of ten to the pilot’s cardiac problems. Toxicology revealed opioid pain medication in four accident pilots.

## 4. Discussion

In this study, with strict inclusion criteria, 5% of fatal aviation accidents in general aviation were estimated to be related to the pilot’s unreported health issues in the U.S. during 2015. This figure probably underestimates the cases and indicates the minimum frequency of this problem. Partially impaired performance related to medicine use is potentially greater than what an accident investigation can prove based on postmortem toxicology [14].

Safety investigations of fatal accidents, medical records and toxicological analyses may reveal that a pilot was unfit because of some health issue related to treated illnesses or injuries and/or prescribed medications, and the current health status was not compatible with flying before the accident. To increase aviation safety and meet the rare situations of an unfit pilot not willing to inform the local civil aviation authority, many countries have adopted legislation regarding a doctor’s duty of notification and in the U.S., more specifically for AMEs [15].

### 4.1. Medications and Conditions

In the recent study of 6677 pilots fatally injured in accidents in U.S. between 1990 and 2012, there were generally increasing trends among pilots’ use of potentially impairing drugs and drugs for potentially impairing conditions [11]. According to this report, recreational and use of, for instance, marijuana products warrants attention. Among pilots, psychotropic medication may sometimes independently form symptoms and functional impairment, affecting fitness to fly due to possible side effects, while evidence-based psychosocial interventions offer accessible treatment options. Selective SSRIs, such as fluoxetine, escitalopram, sertraline or citalopram, may have a waiver in the U.S., through a specific acceptance protocol defined by the FAA [13,16]. Otherwise, antidepressant medication is incompatible with flying in the U.S.

Prescription stimulants are first-line pharmacological treatments for attention deficit hyperactivity disorder (ADHD) but may also be used inappropriately for other purposes [17]. However, diagnosed ADHD requires thorough aeromedical evaluation before fitness to fly—assessment and serious ongoing symptoms are not compatible with flying [18,19]. Civil aviation authorities regard severe long-term psychiatric disorders (e.g., psychotic disorders, bipolar disorder and complicated substance dependence disorders) in general as disqualifying. Otherwise long-term assessment of fitness to fly can only be made after the psychiatric diagnoses are carefully assessed, effects of treatment on symptoms and long-term functioning are followed, rehabilitation is completed and the success of treatment is thoroughly evaluated. Special attention is warranted in conditions where the prognosis varies widely from full recovery to long-term dysfunctional conditions, including major depressive disorders and posttraumatic stress disorders.

Case series studies of pilot aircraft-assisted suicides [20], bipolar disorder [21], and ADHD [19] in fatal aviation accidents suggest that the AME sometimes has only limited access to all relevant health care information. A similar observation, concerning pilots over 70 years old who had had psychotropic medication preceding the fatal accidents, was found in 7% (7/114) of toxicological analyses [14]. The Civil Aviation Safety of Australia (CASA) advises to notify any psychiatric condition requiring psychotropic medication, any condition treated with psychotropic medication and any incident or accident related to alcohol or other drugs [22]. The pilot in the first instance should provide notification. This is in line with the Pilot Fitness Aviation Rulemaking Committee report, which emphasized education, lowering barriers to care and clarification of duty of notification [3].

### 4.2. Timing and Decision Process in Duty of Notification

The issue of timing is about the duration of risk exposure. Civil aviation rules are written so that when risks are foreseen it is appropriate and timely to mitigate those risks. A treating doctor may be an uninformed third-party risk acceptor when receiving a non-disclosure request from a patient.

When collecting data, an AME takes account of privacy rules regarding disclosing information about a patient to an authority at every consultation. To follow these rules, an AME considers why the information was collected and whether the information was provided voluntarily. Additionally, a medical professional should evaluate ‘reasonable belief’ and consider the consequences of disclosure. A doctor should always discuss notification with the patient if at all possible and limit the disclosure of information to an absolute minimum.

**Figure 1:**
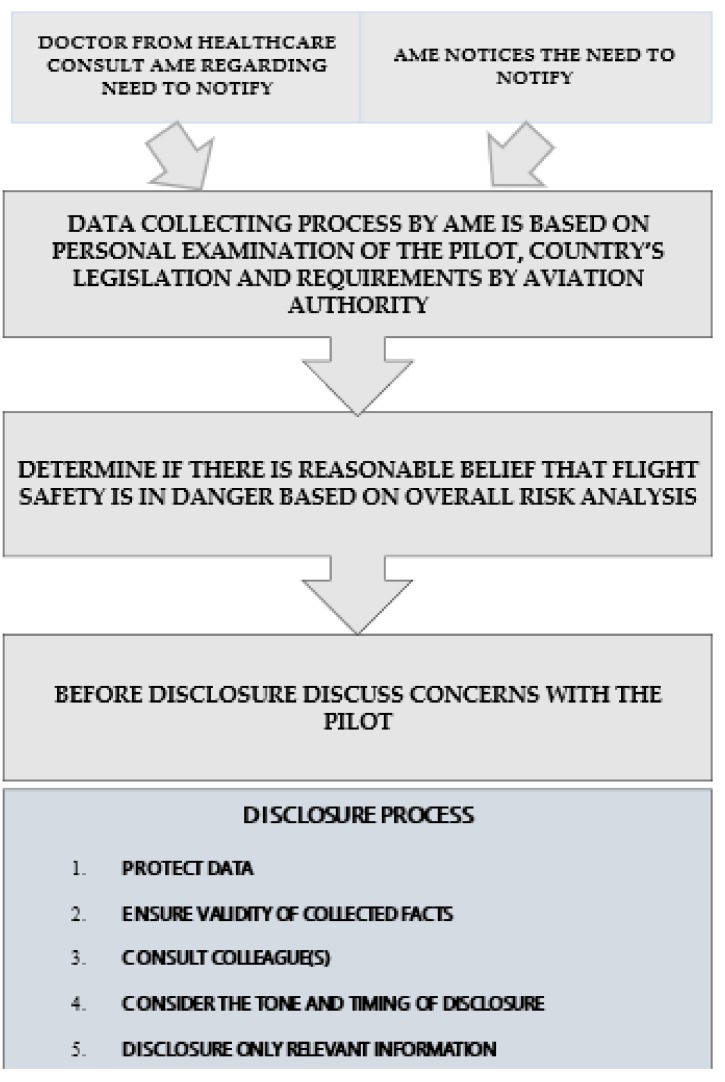
The key elements in the process of data collecting and disclosure.

It is advisable to follow a defined process and not try to decide intuitively or ad hoc whether a doctor has a duty to notify in a specific case. The key elements of the process are described in Figure 1. This figure is proposed by the authors and is based on the practice in New Zealand [4]. Sometimes, a doctor/AME may believe that it is too early to use duty of notification, and a pilot is not willing to follow the obligation. In this situation, the health issue may be monitored. There is a risk in these cases, however, that duty of notification will be delayed. The “drift to failure” theory postulated by Vaughan [23] states that over time, if people accept deviations, they start to treat these deviations as the norm. Based on practical experience in the context of the pilot and AME/doctor relationship, this “drift” can translate into a situation where when the disease gets worse, there is a possibility that these symptoms will reach unacceptable levels. Therefore, in the medical context, it is important to use duty of notification at the earliest stage feasible when the medication or conditions can compromise flight safety to avoid unnecessary delay. There will be times when there is inadequate information to ascertain the aeromedical significance of a health condition, but once the doctor/AME has enough information (s)he should act. If the pilot does not agree with the AME/doctor’s opinion regarding his flight fitness and if the AME/doctor does not act on this problem, the pilot could seek a more suitable opinion of his performance from a different AME/doctor. Maybe the most comprehensive analysis of such a situation is that of the Germanwings pilot. Before the incident, he sought help for his treatment from several doctors [7]. It can be argued that in this case a pilot caused a complex situation in health care organization. This is especially because of the lack of comprehensive coordination in German regulations on when a threat to public safety outweighs the requirements of medical confidentiality.

### 4.3. Duty of Notification as a Part of Safety Management System (SMS)

The mitigation of public safety risks is rarely the responsibility of a single layer of governance and risk mitigation. Governments have a role in ensuring that the medical certification system is sufficiently robust to detect flight safety risks from medical impairment or incapacitation. This responsibility might include data matching to prescription monitoring program and impaired driving or national health databases for pilots. Alternatively, AMEs could be required to consult with a pilot’s general practitioner when issuing a medical certificate to check for medical history and medication information. The Civil Aviation Authority should also be accessible to concerned doctors or lay people for advice on the possible impact of medical conditions or medication on flight safety.

Since 2009, the International Civil Aviation Organization (ICAO) has required that airlines have a safety management system (SMS) [24]. Based on the above mandate, aviation industry organizations include SMS systems to detect and manage undisclosed disease incidents as soon as they arise, including management, pilot education and confidential disclosure systems.

Currently, an advanced SMS system is performance-based and interactive [25]. The aim is to safely manage the complex aviation system in real time. The challenge is to incorporate real-time data regarding possible health changes in the airline’s interactive SMS system. The integration of duty of notification as a part of SMS would help to achieve this target.

### 4.4. Implications for the Notification System

Disclosure of medical information irrespective of a pilot is always a complex issue, in which balance between pilot privacy and public safety should be weighed [26]. Absolute confidentiality may give too much emphasis to individual autonomy [27] and may result in harm to the public, as well as to the pilot. As legislation is enacted related to medical confidentiality, it is desirable that the complexity of this task does not discourage the legislator from creating applicable law [28]. The recommendation to develop clear rules for the duty of notification process, which was presented by BEA, and the Pilot Fitness Aviation Rulemaking Committee report are supported. Regarding aviation, especially commercial aviation, internationally accepted notification processes could be potentially useful.

### 4.5. Limitations

The major limitation of this study is that the pre-accident health data is based solely on the information provided in the accident reports. The availability of comprehensive medical record information varied between the accident reports. Additionally, the accident cases where mostly (8/10) related to general aviation.

## 5. Conclusions

Professional guidance defining the duty of notification process is urgently needed. Following the proposed process related to key elements in data collecting and disclosure may help a doctor/AME achieve the necessary balance in this decision. The process of notification of authorities without a pilot’s express permission needs to be carried out by using a carefully planned protocol that works within legislation and professional responsibilities to address the pilot and the public, as well as the healthcare provider. Our results show that active but balanced use of a doctor’s duty of notification could potentially improve flight safety.

## Figures and Tables

**Table 1:** Duty of notification policies in different countries.

Country	Policy
Norway	Duty of notification covering all doctors since 1982
New Zealand	Duty of notification covering all doctors since 2003. Practical protocol created and implemented
Finland	Bill of reform that duty of notification will cover all doctors
United States	Notification responsibilities vary state by state

**Table 2:** Fatal aviation accidents where National Transportation Safety Board (NTSB) cause or contributing cause of accident is related to pilot health issues not reported to an aeromedical examiner in 2015 *.

Accident Date, State, Pilot Age (Years), Gender (M = Male), No. of Deceased	Last Medical Certification and Comments *	Reason of Incompatibility with Flying	NTSB Cause of Accident
11 December 2015, PA 68, M 3	02/22/2014 Class 3	Amphetamine	The pilot’s failure to maintain control of the airplane after a cabin door came open in flight. *Contributing to the accident was the pilot’s misuse of amphetamine.*
6 December 2015, MO 40, M 2	05/26/2012 Class 3 Convictions for driving under the influence, possession of a controlled substance thrice, reported last using methamphetamines 2011.	Methamphetamine	The non-instrument-rated pilot’s loss of control due to spatial disorientation. *Contributing to the accident was the pilot’s use of methamphetamine, which impaired his decision-making abilities.*
18 November 2015, CA 65, M 2	01/19/2015 Class 3 Pilot reported hypertension and the use of medications including nebivolol, pantoprazole and rosuvastatin.	Alprazolam diphenhydramine	The pilot’s loss of control during landing on a dolly. Contributing were the pilot’s decision to conduct the flight without an instructor despite recommendations, failure to land on the ramp when there was difficulty landing on the dolly, and *his impaired decision-making, judgment, and psychomotor performance, due to use of two psychoactive drugs.*
9 November 2015, GA 40, M 2	05/07/2013 Class 3	Amphetamine (possible medical use), tramadol, cetirizine, doxylamine, marihuana, ethanol ingested or post-mortem	The pilot’s loss of airplane control due to spatial disorientation. *Also causal to the accident was the pilot’s impairment by the combined effects of multiple medications and drugs.*
9 November 2015, CO 63, M 2	11/04/2013 Class 3 Pilot reported the use of rosuvastatin and niacin.	Sertraline, diphenhydramine, cetirizine, marihuana	The pilot’s loss of airplane control in high density altitude conditions, which resulted in an inadvertent stall. *Contributing was the pilot’s impaired performance due to his use of potentially impairing medications.*
17 October 2015, CA 71, M 2	01/01/2014 Class 3 Pilot reported hypertension treated with atenolol and amlodipine	Quetiapine, hydrocodone	The non-instrument-rated pilot’s improper inflight decision-making to attempt to outclimb clouds along his planned route, which resulted in his inadvertent entry into instrument meteorological conditions, spatial disorientation, and a loss of control. *The pilot’s preexisting medical conditions and his use of impairing medications contributed to his degraded performance.*
2 September 2015, TN 66, M 1	06/10/2015 Class 2	Autopsy: scarring due to recent heart attacks.	*The pilot’s incapacitation from complications of a recent heart attack*, which resulted in a loss of control during cruise flight.
16 August 2015, NY 59, M 1	12/22/2014 Class 2	Amphetamine (possible medical use), oxycodone oxymorphone, clonazepam	Decision to delay turning after engine failure, and controller provided erroneous info. Contributing were (1) the FAA’s lack of requirement to validate radar video maps, (2) the failure of the engine crankshaft due to a bearing shift, (3) *the pilot’s impairment due to amphetamine abuse and underlying medical condition(s).*
1 August 2015, AR 38 M 1	01/23/2015 Class 2 Pilot reported a previous eye problem, surgery, and earlier hospitalization related to a vehicle crash	Buprenorfine, diphenhydramine	The pilot’s inability to maintain control of the airplane as a result of *incapacitation by drug effects or an acute cardiac event.*
4 July 2015, TX 28, M 2	07/18/2014 Class 2	Alprazolam, hydrocodone, ethanol, evidence on cocaine withdrawal	The pilot’s low-altitude aerobatic display, which resulted in an aerodynamic stall/spin when he exceeded the airplane’s critical angle of attack. *Contributing to the accident was the pilot’s impairment due to alcohol and drugs.*

* Based on medical information in the accident investigation.
